# The Emerging Function and Promise of tRNA-Derived Small RNAs in Cancer

**DOI:** 10.7150/jca.89219

**Published:** 2024-01-27

**Authors:** Na Yang, Ruijun Li, Ruai Liu, Shengjie Yang, Yi Zhao, Wei Xiong, Lu Qiu

**Affiliations:** 1 College of Resources, Environment and Chemistry, Chuxiong Normal University, Chuxiong 675000, China.; 2 College of Basic Medical Sciences, Dali University, Dali 671000, China.; 3 College of Foreign Languages, Chuxiong Normal University, Chuxiong 675000, China.; 4 The People's Hospital of ChuXiong Yi Autonomous Prefecture, Chuxiong 675000, China.

**Keywords:** tsRNAs, tRF, tiRNA, cancer, molecular mechanisms

## Abstract

Fragments derived from tRNA, called tRNA-derived small RNAs (tsRNAs), have attracted widespread attention in the past decade. tsRNAs are widespread in prokaryotic and eukaryotic transcriptome, which contains two main types, tRNA-derived fragments (tRFs) and tRNA-derived stress-inducing RNA (tiRNAs), derived from the precursor tRNAs or mature tRNAs. According to differences in the cleavage position, tRFs can be divided into tRF-1, tRF-2, tRF-3, tRF-5, and i-tRF, whereas tiRNAs can be divided into 5'-tiRNA and 3'-tiRNA. Studies have found that tRFs and tiRNAs are abnormally expressed in a variety of human malignant tumors, promote or inhibit the proliferation and apoptosis of cancer cells by regulating the expression of oncogene, and play an important role in the aggressive metastasis and progression of tumors. This article reviews the biological origins of various tsRNAs, introduces their functions and new concepts of related mechanisms, and focuses on the molecular mechanisms of tsRNAs in cancer, including breast cancer, prostate cancer, colorectal cancer, lung cancer, b-cell lymphoma, and chronic lymphoma cell leukemia. Lastly, this article puts forward some unresolved problems and future research prospects.

## Introduction

Generally, tRNAs are defined as adaptor molecules that help ribosomes decode messenger RNA (mRNA) and deliver amino acids to the ribosome during protein biosynthesis^1^,^2^. They have the secondary structure of the clover model, which consist of three rings, namely the D ring, the anticodon ring, and the TΨC ring; four stems, namely D stem (stem connected with D ring), anticodon stem (connected with anticodon ring), TΨC stem (connected with TΨC ring) and amino acid receiving stem; and the variable arm between the anticodon stem and the TΨC stem. After using RNA sequencing technology and some new analysis tools to analyze tRNA, it was found that most of the modification components in nucleic acid are found in tRNA^3^. The diversity of tRNA genes is due to the diversity of gene duplication and tRNA isode coder(tRNAs share the same anticodon, but have differences in RNA sequences) and the diversity of isoacceptors (tRNAs with different anticodons, but capable of transporting the same amino acid)^4^.

Noncoding RNA refers to RNA that does not encode protein^5^. High-throughput transcription analysis has shown that eukaryotic genomes have transcribed up to 90% of genomic DNA, only 1⁓3% of transcripts encode proteins, and the vast majority are transcribed as noncoding RNAs (ncRNAs), which includes two categories: basic RNA, with transferring RNAs (tRNAs) and ribosomal RNA (rRNAs), and regulatory RNA, with long noncoding RNAs (lncRNAs) and small noncoding RNAs (sncRNA)^6^,^7^. Among sncRNAs, the roles of microRNAs (miRNAs), endogenous siRNAs (endosiRNAs) and pivi-interaction RNAs (piRNAs) in cancer have been well verified^8^,^9^. miRNAs, which exert biological functions through posttranscriptional regulation of gene expression in many human diseases such as cancer, have especially been the focus of recent attention^10^-^12^. In recent years, with the development of deep sequencing technology, many more classes of sncRNAs have been discovered.

These tRNA-derived sncRNAs are called tsRNAs. tsRNAs are not random tRNA degradation products^13^-^15^. In fact, the biogenesis of tsRNAs is controlled by a set of highly conserved and precise site-specific cleavage mechanisms, which can produce 14⁓50 nucleotides in length^16^. It has been reported that tRFs are present in such cancers as breast, lung, and colorectal cancers. This indicates that tsRNAs may play an important role in tumorigenesis. The complex molecular mechanism of tumor occurrence and development has always been the focus of cancer research. In the case of insufficient blood supply, cancer cells can only grow in a microenvironment lacking oxygen and limited nutrients. Cancer cells can adapt to this stressful environment through different regulatory strategies to ensure survival and proliferation^17^. The tsRNAs produced by tRNAs under stress is one of the important ways. Therefore, we have reason to believe that tsRNAs must be closely related to cancer treatment. In addition, there is increasing evidence that tsRNAs can be detected in the urine and serum of cancer patients and can be used as molecular markers for tumor diagnosis^18^-^20^. Here, we will review the biogenesis and biological functions of tRNA-derived small RNAs. According to common cancer types such as breast cancer and prostate cancer, the current understanding of the molecular mechanism of tsRNAs in tumor pathogenesis is discussed. Finally, we will explore the potential of using tsRNAs as clinical biomarkers for cancer diagnosis and prognosis as well as therapeutic targets for cancer treatment.

## Biogenesis and classification of tRNA-derived small RNAs

After eukaryotic tRNA is encoded by a large number of genes, it is first transcribed by RNA polymerase III in the nucleus and evolved into a transcript with a typical clover structure^21^. The clover structure contains a 5'-leader sequence and 3'-tailer sequence, which are respectively excised by ribonuclease P (RNase P) and ribonuclease Z (RNase Z, ELAC2) and then catalyzed by tRNA nucleotide transferase 1 (TRNT1). The terminal cytosine-cytosine-adenosine (CCA) trinucleotide is added to all mature tRNAs, which is a necessary condition for aminoacylation^22^. Precursor tRNAs and mature tRNAs are specifically modified in the nucleus and cytoplasm, respectively, to produce different tRNA-derived fragments. According to the position of cleavage, these fragments can be divided into different types.

tRF is the most abundant small noncoding RNA in serum^23^. After analyzing the published small RNA data of mice^24^, *Drosophila melanogaster*^25^, *Caenorhabditis elegans*^26^, and fission yeast^27^, it was found that tRF-5s and tRF-3s can be seen in all species, but not all tRNAs produce tRF, and the expression of tRF in different species is uneven. In general, the abundance of tRF-5s is higher than that of tRF-3s, and the abundance of tRF-3s is higher than that of tRF-1s^28^. tRNA is expressed in a tissue-specific manner. The length, starting and ending points, and relative abundance of tRNA fragments depend on gender, population, tissue, disease, and disease subtype^29^. The expression of tRFs and tiRNAs in different biological fluids (including saliva, serum, amniotic fluid, semen, urine, and bile) is quite different^30^. tRFs and tiRNAs are modified and not easily degraded, so they are more stable than linear RNA^31^, but the stability between different fragments may be asymmetric.

### tRNA-derived fragments (tRFs)

The length of tRF is approximately 14 nucleotides (nt)-30 nt^32^. According to the biogenesis and length is divided into the following subtypes: tRF-5, tRF-3, tRF-2, tRF-1, and i-tRF. tRF-5 is generated from the 5' end of the mature tRNA. Moreover, it is produced by cutting the D-loop or the arm stem between the anticodon loop and D-loop, and this process mainly relies on the Dicer enzyme^33^. tRF-5 has a 5' phosphate, which is mainly found in the nucleus. Given the different cutting sites, tRF-5 can be divided into the following three subtypes: (1) tRF-5a (14-16 nt), produced by cutting D-loop; (2) tRF-5b (22⁓24 nt),generated by cutting the D-stem; and (3) tRF-5c (28⁓30 nt), formed by cutting the anticodon stem^34^. These subtypes differ only in length and share a common seed sequence. Analogously, tRF-3 derived from the 3' end of the mature tRNA is produced by cleavage of ANG, Dicer, or members of the ribonuclease A superfamily at the T-loop. Therefore, there is a 3' hydroxyl, mainly discovered in the cytoplasm, containing the CCA sequence in the tRF-3 tail. There are two distinct tRF-3 types corresponding to the same tRNA: tRF-3a and tRF-3b. The length of 18 nt is defined as tRF-3a, whereas the length of 22 nt is tRF-3b^34^. Unlike tRF-5, tRF-3a and tRF-3b have different 5' ends, so their seed sequences are completely different^35^.

tRF-2 is induced under hypoxic conditions and produced by cleavage of the anticodon loop of tRNA. It only covers the anticodon stem and loop regions and does not contain the typical 5' end and 3' end structures^36^,^37^. tRF-2 is a newly discovered tRF derived from tRNAGlu, tRNAAsp, tRNAGly, and tRNATyr^38^. tRF-1 is derived from the 3' end of precursor tRNA cleaved by RNase Z or its cytoplasmic homolog ribonuclease Z 2 (ELAC2)^39^. The 3' end of tRF-1 contains a poly U sequence, so it is also called 3' U-tRF^40^. i-tRF is derived from the internal region of any mature tRNA, excluding the terminal regions of 5' and 3'. i-tRF is named based on the starting position of the 5' end in the tRNA. According to the starting position of the 5' end in the tRNA, the corresponding i-tRF can be named. For example, the fragment produced by cutting at the anticodon loop is named A-tRF, V-tRF refers to the fragment produced by cutting in the variable region, and D-tRF is the fragment produced by cutting in the D-loop^41^.

### tRNA halves (tiRNA)

tiRNA is produced by cleaving the anticodon loop of mature tRNA 31⁓40 nt in length, although it also can be divided into 5'-tiRNA and 3'-tiRNA according to whether the 5' end or 3' end sequence contains anticodon splicing sites. 5'-tiRNAs start from the 5' end of the mature tRNA to the end of the anticodon loop; 3'-tiRNAs start from the anticodon loop to the 3' end of the mature tRNA^42^. tiRNA is produced under certain stress conditions, such as phosphate deficiency, amino acid deficiency, heat shock, ultraviolet radiation, hypoxia, oxidative stress, and viral infections^43^. Although tiRNAs were originally named as stress fragments, other types of tRNA halves can also be detected under non-stress conditions^44^. tiRNAs can also be found under specific non-stress conditions, showing that biological processes other than cellular stress responses may be regulated by tiRNAs^45^. Some modifications in transfer RNAs are essential for RNA structure and function^46^. Meanwhile, tiRNA production can be affected by the modification of tRNA^47^. The stress-induced cleavage of tRNAs depends on Dnmt2, and, more importantly, Dnmt2-mediated methylation protects tRNAs from ribonuclease degradation^48^. In addition, studies have shown that the production of tiRNAs is closely related to angiogenin (ANG), whether in vitro or in vivo^49^. However, it was subsequently suggested that ANG is not the only RNase that produces tiRNA^50^. In short, many factors, such as the kind of stress, angiogenin availability and activity, tRNA substrate level, and global protein synthesis rates, can influence the production of tiRNAs^51^.

## Biological function of tRNA-derived small RNAs

Information about the functions of tRFs and tiRNAs in cells is constantly increasing. However, most studies only describe the expression of tRFs and tiRNAs in human cell lines, but their expression levels in human tissues are still unclear. tRFs and tiRNAs and microRNAs (miRNAs) have similar functions^52^. For example, the sequences of miR-1280, miR-1274a/b, and miR-886-5p are the same as the 3'-end of tRNALeu, the 3'-end of tRNALys, and the 5'-end of tRNAAla, respectively^30^. In addition, tRFs are generated in a way that depends on Dicer^53^. Studies have confirmed that tRFs act as miRNAs in Drosophila.tRF-5 and tRF-3 have typical seed regions and can form tRF-miRNA chimeras^54^. Through bioinformatics analysis, an obvious positive correlation between miR-1274-a/b and tRNALys3/tRNALys5 is apparent, suggesting that these two miRNAs are essentially tRFs^55^,^56^. Therefore, tRF and tiRNA can perform biological functions in a similar way to miRNA.

### Regulation of RNA Reverse Transcription

tsRNAs can be used as guide RNA for reverse transcription of viral RNA. The host cell tRF-3 (abbreviated as tRF-3019) is derived from the 3' ends of tRNA-Pro and can bind to the primer binding site (PBS) of human t-cell leukemia virus type 1 (HTLV-1) RNA. It has been confirmed that tRF-3019 can induce HTLV-1 reverse transcriptase, thereby initiating reverse transcription, promoting the self-synthesis of the virus, and becoming a potential new target for controlling HTLV-1 infection^57^. At the same time, respiratory syncytial virus (RSV) can induce tRF5-GluCTC to promote the replication of RSV virus, thereby triggering the stress response of host cells. The 3'end of tRF5-GluCTC inhibits its expression by recognizing a target in the 3'-untranslated region of apolipoprotein E receptor 2 (APOER2). APOER2 is an anti-RSV protein, and the inhibition of APOER2 by tRF5-GluCTC can promote RSV replication. In short, tRFs are important molecules that control RSV replication. These studies provide a potential therapeutic target for controlling RSV replication by regulating tRF induction^58^-^60^. Therefore, both tsRNAs can be used as primers for reverse transcription.

### Regulation of translation

Compared with other noncoding RNAs, tsRNAs inhibit or activate protein synthesis through some unique mechanisms. They inhibit overall translation by substituting translation initiation factors, or combine with ribosome-related aminoacyl-tRNAsynthetases (aa-RSs) and inhibit in vitro translation by affecting the aminoacylation of tRNA^61^-^64^. In addition, tRF also binds to small ribosomal subunits. For example, the result of Val-tRF binding is to displace mRNA from the initiation complex, which leads to a decrease in overall translation in vivo and in vitro^65^. Lu's research revealed that global translation is regulated by 5' tsRNAs by suppressing the mRNA translation of ribosomal proteins (RPs), eukaryotic translation initiation factors (eIFs), or eukaryotic translation elongation factors (eEFs) in Drosophila^66^.

Angiopoietin (ANG) is a secreted ribonuclease that can cleave tRNA and initiate the stress response of mammalian cells. Studies have found that ANG can inhibit protein synthesis and promote the assembly of stress granules (SGs)^67^. Knockout of the angiogenin inhibitor RNH1 can enhance the production of tiRNA and promote arsenous acid-induced translation block^68^. These findings indicated that SG assembly is part of the stress response induced by ANG and tiRNA.

In most cases, stress-induced phosphorylation of the translation initiation factor eIF2 induces the assembly of stress granules by preventing or delaying translation initiation. Phosphorylation of eIF2α reduces the availability of the eIF2α-GTP-tiRNAMet ternary complex required for translation initiation, leading to translation stagnation^69^. Simultaneously, translation inhibition mediated by tiRNA has also been verified by replacing the eIFs 4B, 4E, and 4G in the mRNA m7G cap. Among them, eIF4G is the main scaffold protein in the translation initiation complex and directly binds to tetrameric G-quadruplex (G4) structures^70^. The conserved residues of 5' tRFs can inhibit protein translation. This effect does not require any complementary targets in the reporter gene sequence but requires a generally conserved "GG" dinucleotide in tRF^71^.

tRFs can also regulate translation by interacting with ribosomes. Studies have found that a specific LeuCAG 3' tsRNA could bind to the coding and noncoding 3'-UTR sequences in ribosomal protein S28 (RPS28) mRNA, thereby enhancing its translation and ultimately increasing the number of ribosomes^72^. RPS28 is necessary for 18S rRNA biosynthesis. Moreover, RPS28 is a component of the 40S ribosomal subunit. Therefore, the expression of RPS28 can be reduced by inhibiting LeuCAG 3' tsRNA and thereby damaging the 18S rRNA pathway, thus reducing the survival ability of cancer cells and promoting apoptosis of cancer cells. Studies have also confirmed that inhibiting LeuCAG 3' tsRNA can destroy the occurrence of ribosomal biology, which in turn leads to decreased cell viability and apoptosis of cancer cells such as HeLa and HCT-116 (human cervical cancer cell line and colon cancer cell line)^73^,^74^.

### Regulation of gene expression

The expression of tRNA genes is specific to cell type and tissue. In addition, about half of human tRNA genes are silent or low-expressed, so the abundance of tsRNAs varies in different cells and tissues^75^. When tsRNAs are processed and accumulated in the nucleus, they are subsequently exported to the cytoplasm, which indicates that tsRNAs can regulate gene expression at different levels^39^. In several organisms, tRFs and tiRNA regulate gene expression like miRNA through interactions with Argonautes proteins. Ago protein is an essential effector protein in all gene silencing pathways guided by small RNAs^76^,^77^.

RNA interference (RNAi) is an effective gene-silencing mechanism; it reveals the unique ability to target cancer-related genes. Because of this, many oncogene products related to tumorigenesis have been increasingly studied as possible targets for new treatments based on RNAi strategies. In the process of RNAi regulating gene silencing, Ago and Dicer are the main participants^78^. Yeung et al. found that the 18-nt tRF derived from tRNALys is highly expressed in human immunodeficiency virus type 1 (HIV-1), which is called tRF-3006^79^. In the liver cancer cell line Huh7, researchers have discovered a unique set of tRFs, which are derived from the 3' trailers of the pre-tRNA. Among them, tRF_U3_1 is most expressed in the Huh7 cell line and cancerous liver tissues. tRFs derived from a large number of pre-tRNA trailers (tRF_U3_1) can be stabilized by binding La/SSB to their 3' U-tail and negatively regulate La/SSB-dependent viral gene expression^80^. In addition, compared with AGO2, tRF-5 is more inclined to bind AGO1, which is related to posttranscriptional RNA silencing^81^.

### Regulation of the cell cycle

According to reports, some tRFs and tiRNAs can bind to cytochrome C (Cyt C) and block the activation of caspase-9, thereby inhibiting the formation of apoptotic bodies and ultimately promoting cell survival^82^. In normal cells, tRFs act as endogenous apoptotic signals, inhibiting the regulatory factors of related apoptotic proteins, and directly or indirectly cause cell apoptosis. When cells are under stress, tRFs increase significantly, deregulate the process of apoptosis, and induce the proliferation of malignant cells^83^. Another study found that tRNAguanosine 9-hypomethylation caused tRNAGln fragment fragmentation and 5'-tRNAGln fragment mediated cell death caused by TRMT10A deletion^84^.

### Regulation of intergenerational inheritance

Intergenerational inheritance refers to the transmission of an epigenetic feature from one generation to the next^85^. Several tRFs and tiRNAs have been found to act as epigenetic regulators. The role of tsRNAs in intergenerational inheritance was confirmed for the first time in a study of feeding mice, which showed that sperm tsRNAs can help pass acquired metabolic disorders to the next generation^86^. Inutero, malnutrition changes sperm DNA methylation in F1 male mice and affects the metabolism of F2 mice^87^. In addition to the mother's condition, the father's diet also affects the offspring's metabolism^88^,^89^. Eating a high-fat diet (HFD) can cause b-cell dysfunction in male rats and glucose intolerance in their female offspring. The offspring of male mice fed a low protein diet (LPD) also showed increased expression of genes related to lipid and cholesterol biosynthesis in the liver.

## Molecular mechanism of tRNA-derived small RNAs in cancer

Several pieces of evidence have supported that sncRNAs, including miRNAs and Piwi-interacting RNAs (piRNAs)^90^,^91^, have been associated with the occurrence, progression, and drug response of cancer. In solid tumors, the continuous and rapid growth of cancer cells will exceed the supply of nutrients and oxygen, resulting in a hypoxic environment^92^. Angiopoietin is a tumor angiogenic factor that stimulates the development and progression of tumors under hypoxic conditions. Angiopoietin is reported to be up-regulated in many solid tumors^93^. Angiopoietin not only plays a role in angiogenesis, but also cleaves mature tRNAs into tiRNAs. So far, 232 annotated tsRNAs have been included in the Cancer Genome Atlas (TCGA) and NCI-60 cell line screening database^94^, but this number will increase.

### Breast cancer

Breast cancer is a hormone-dependent tumor, and most researchers believe that the increase in its incidence is related to estrogen^121^. A new type of tRNA-derived small RNA called Sex Hormone-dependent tRNA-derived RNAs (SHOT-RNAs) is specifically expressed in large quantities in sex hormone-dependent breast cancers. SHOT-RNAs are produced by aminoacylated mature tRNAs through angiogenin-mediated anticodon cleavage, which is promoted by estrogen and its receptors^99^. tRF3E is 3-tRF derived from tRNAGluTTC, which is expressed in the healthy breast but not in breast cancer (BC) tissue. The level of tRF3E in the serum of her2-positive BC patients gradually decreases during the development of the tumor and reaches the lowest value in the metastatic state. As a tumor suppressor, tRF3E competes with the over-expressed RNA binding protein nucleolin (NCL) in BC to cause the release of p53 mRNA, which in turn promotes the translation of p53, thereby inhibiting the proliferation of cancer cells^95^. Triple negative breast cancer (TNBC) is associated with a hypoxic phenotype, and hypoxia contributes to the chemotherapy resistance of breast cancer. Studies have found that in TNBC cells stimulated by hypoxia, tDR 0009 and tDR 7336 are significantly upregulated and promote the resistance of TNBC cells to adriamycin^96^. Research data shows that tsRNA-26576 is upregulated in breast cancer and tsRNA-26576 can be used as an oncoprotein to inhibit the expression of SPEN and FAT4, which indicates that it may act as a potential tumor-initiating factor to promote the proliferation, migration, and migration of cancer cells. Play an important role in invasion and inhibition of cell apoptosis^97^.

Similarly, a protein-dependent replacement mechanism was also confirmed in a previous study after induction by replacing 3'UTRs from the Y-box-binding protein 1 (YBX1), which inhibited the stability of multiple oncogenic transcripts in breast cancer cells^37^. YBX1 is a multifunctional DNA/RNA binding protein that binds to 5'-tiRNA and mediates translation inhibition and stress granule formation^61^. The unique G-quadruplex structure of 5'-tiRNA is the key to inhibitting translation by binding YBX1^62^. This further shows that some tRFs are involved in the regulation of posttranscriptional gene expression. Compared with normal controls (NCs), six tRFs in plasma samples of early-stage breast cancer (EBC) patients were significantly downregulated, including tRF-Glu-CTC-003, tRF-Gly-CCC-007, tRF-Gly-CCC-008, tRF-Leu-CAA-003, tRF-Ser-TGA-001, and tRF-Ser-TGA-002^122^. Studies have explored whether and how the interaction between tRF expression and T cell activation affects the survival of breast cancer patients. For example, the expression levels of tRFdb-5024a, 5P_tRNA-Leu-CAA-4-1, and ts-49 were positively correlated with overall survival, whereas the expression levels of ts-34 and ts-58 were negatively correlated^123^. Koi et al. used miR-21-5p (3 'addC), miR-23a-3p and tRF-Lys (TTT) in the diagnosis of BC, with a high accuracy (AUC = 0.92), and distinguished BC from the control group, indicating that they can be used as biomarkers for breast cancer diagnosis 124.

The sncRNA in the blood circulation can act as a signal molecule to perform various cellular functions. For example, early studies have shown that the expression levels of 5'-tiRNA from tRNA-Arg, -Asn, -Cys, -Gln, -Gly, -Leu, -Ser, -Trp, and -Val in the circulation of breast cancer patients are significantly increased, and the expression level of 5'-tiRNA from tRNA-Asp and tRNA-Lys is reduced^125^. Then Mo et al. found that AS-tDR-001430 was derived from a 5'-half fragment of tRNA-Val-CAC, also called as 5'-tiRNAVal. 5'-tiRNAVal expressed significantly low in breast cancer tissues and inhibited the translation of FZD3 by directly targeting the 3'-UTR of the human Frizzled homolog 3 (FZD3). 5'-tiRNAVal suppressed cells' malignant activities by inhibiting the FZD3/Wnt/β-Catenin signaling pathway^98^. Balatti et al. conducted a statistical analysis of oncogenes at different developmental stages in normal breast epithelial cells and BC cells with activating mutations of oncogenes and found that oncogenes can regulate tsRNAs. For example, ts-3 is significantly downregulated in advanced aggressive BC cells, whereas ts-6, ts-48, and ts-67 are upregulated in advanced BC cell lines^107^. Studies have confirmed that RUNX1 can directly and/or indirectly regulate tsRNA to inhibit BC phenotype. Because ts112 is the only tsRNA that can be significantly downregulated after RUNX1 overexpression, it is subsequently confirmed that ts-112 is necessary to enhance the proliferation of BC cells and promote the proliferation of normal breast epithelial cells^100^. Moreover, highly expressed tRF-30-JZOYJE22RR33 and tRF-27-ZDXPHO53KSN in trastuzumab-resistant BC are associated with poor progression-free survival, so they may be potential biomarkers and intervention targets for clinical treatment of trastuzumab BC point^101^.

The above analysis shows that tsRNAs are the most widely reported in BC, and the mechanism research is the most in-depth. tRF3E, **ts**RNA-26576, tRF-Glu-CTC-003, tRF-Gly-CCC-007, tRF-Gly-CCC-008, tRF-Leu-CAA-003, tRF-Ser-TGA-001, tRF-Ser-TGA-002, tRFdb- 5024a, 5P_tRNA-Leu-CAA-4-1, ts-49, tRF-Lys (TTT) and 5'-tiRNAVal are expected to be biomarkers for BC diagnosis. tRF-30-JZOYJE22RR33, tRF-27-ZDXPHO53KSN are expected to be potential targets for BC therapy.

### Colorectal cancer

As mentioned earlier, tRF-1001 is also closely related to the proliferation of colon cancer cells. In the HCT-116 cell line, after knocking out tRF-1001, the proportion of cells in the G2 phase of the cell cycle increased correspondingly, resulting in a significant decrease in cell survival rate^102^. Subsequently, a study identified 16 differentially expressed tRFs in colon cancer and paired adjacent tissues. At the same time, 55 differentially expressed mRNAs were identified as potential targets of these tRFs and were found to be mainly enriched in the vitamin metabolism pathway and the cyclic guanine monophosphate-protein kinase G signaling pathway^126^. Wu et al. used 5 '-tRF-GlyGCC from plasma as a new diagnostic method for CRC with an AUC as high as 0.882, so 5' -tRF-GlyGCC can be used as a biomarker for CRC 127. Huang et al. reported that tRF/miR-1280, a 17-nt fragment from tRNALeu and pre-miRNA, can directly bind Notch ligand JAG2 in colorectal cancer (CRC) to further inhibit the Notch signaling pathway and reduce tumorigenesis during CRC progression, formation and transfer^104^. As we all know, the Notch signaling pathway is closely related to the proliferation, metastasis, and stem cell-like phenotype of cancer cells^128^,^129^. When the expression of tRF/miR-1280 decreases, the proliferation ability of colorectal cancer cells decreases. More importantly, the inactivation of Notch signal mediated by tRF/miR-1280 suppresses the phenotype of cancer stem cells (CSC)^104^. Studies have reported that ANG is upregulated in CRC tissues and is associated with metastasis in CRC patients. Subsequently, it was found that ANG can promote the growth and metastasis of CRC in both in vivo and in vitro systems. In particular, the expression level of tiRNAs (5'-tiRNA-Val) produced by ANG cleavage is high, and they are enriched in CRC tumor tissues and highly metastatic cells and play a role in CRC metastasis promoted by ANG^105^.

The above analysis shows that there are many studies on tsRNAs in CRC, including mechanism studies. tRF-1001, 5 '-tRF-GlyGCC, tRNALeu, tRNALeu and other tsRNAs are expected to be biomarkers for the diagnosis of CRC.

### Lung cancer

Research by Shao et al. showed that the expression of tRF-Leu-CAG in NSCLC tissues, cell lines, and serum was significantly upregulated and positively correlated with tumor stage. When the expression of tRF-Leu-CAG is inhibited, the expression of Aurora kinase A (AURKA) is also inhibited. This means that tRF-Leu-CAG regulates cell cycle progression by regulating AURKA activity. However, at present, it is not clear whether the AURUK gene is a direct target of TRF-leu-CAG, and previous studies have confirmed that tRF-Leu-CAG can interact with some miRNAs, so AURUK gene expression may be coregulated by both miRNAs and tsRNAs^106^. In addition, compared with paired normal lung tissues, the expression levels of ts-53 and ts-101 (previously named ts-3676 and ts-4521) in lung cancer tissue samples were significantly downregulated. Among them, the downregulation of ts-4521 is related to signal pathways related to cell proliferation and apoptosis. When ts-3676 was overexpressed in lung cancer cell lines A549 and H1299, the colony formation of transfected cells was reduced several times, suggesting that ts-3676 has a tumor suppressor effect^108^. Wang et al. used tRF-16-L85J3KE, tRF-21-RK9P4P9L0 and tRF-16-PSQP4PE to predict lung adenocarcinoma, and the results showed that the AUC in plasma was 0.99 and that in tissue was 0.92 130.

The above analysis shows that there are few studies on tsRNAs in lung cancer, and there are mechanism studies reported. ts-3676, ts-4521, tRF-16-L85J3KE, tRF-21-RK9P4P9L0 and tRF-16-PSQP4PE are expected to be biomarkers for the diagnosis of lung cancer.

### B-cell lymphoma

CU1276 is a Dicer-dependent tRF expressed in mature B cells. It is associated with four Argonautes proteins and can play a role similar to miRNA, inhibiting mRNA transcription through specific sequences. Further studies found that CU1276 is low in lymphoma cell lines and primary biopsies. More importantly, CU1276 can inhibit the endogenous essential gene replication protein A1 (RPA1) involved in DNA dynamics; therefore, CU1276 inhibits the proliferation of lymphoma cells and regulates the DNA damage response by regulating the expression of DNA damage response genes^109^. Guo et al. found the differential expression of tRFs in myelodysplastic syndrome (MDS), especially in predicting patients' response to DNA methyltransferase inhibition therapy. The expression level of tRFs in samples before treatment has important reference value^131^. Subsequent studies have shown that the expression of this tRFs is related to whether the patient will develop acute myeloid leukemia (AML). When the expression level of tRF-Asp in MDS patients is significantly reduced, the patient is likely to progress to AML^132^. These studies confirmed that tDR can be used as a biomarker for the prognosis of MDS. In addition, PI3K/AKT and MAPK/ERK pathways play an important role in the pathogenesis of B-cell lymphoma. Downregulation of neural precursor cell-expressed, developmentally downregulated 4-Like, E3 ubiquitin protein ligase (NEDD4L) can also promote tumor growth and inhibit the MAPK/ERK signaling pathway^133^. Recent studies have shown that AS-tDR-008946 (tRF-3) and AS-tDR-013492 (i-tRF) are both upregulated, which may inhibit the expression of NEDD4 in a miRNA-like manner, thereby accelerating the progression of lymphoma^110^.

The above analysis shows that there are few reports on tsRNAs in B-cell lymphoma, but there are some reports on molecular mechanism. CU1276, AS-tDR-008946 (tRF-3), AS-tDR-013492 (i-tRF) and other biomarkers are expected to be used in the diagnosis of B-cell lymphoma.

### Chronic lymphocytic leukemia

ts-53 (known as miR-3676 in the text) targets the 3'UTR of TCL1, which is a key oncogene for the occurrence of malignant chronic lymphocytic leukemia (CLL), and its downregulation in leukemia cells is negatively correlated with the expression of TCL1^134^.The most downregulated tsRNA in CLL is ts-42, which locates at 15q12 downstream of tRNA11GluTTC. Bioinformatics analysis revealed the existence of a CpG island upstream of tRNA11GluTTC. Similarly, it was found that ts-43 and ts-44 are derived from pre-tRNAHis and are downregulated in CLL samples. Such results suggest that mature tRFs may have carcinogenic and/or tumor suppressor functions in CLL^111^. tRF-3019 perfectly matches the sequence of the primer binding site of human T-cell leukemia virus type 1 (HTLV-1), which is the pathogen of adult T-cell leukemia/lymphoma (ATLL). In vitro reverse transcriptase experiments further confirmed that tRF-3019 can activate HTLV-1 reverse transcriptase, so it may become a new target for controlling HTLV-1 infection^88^.

The above analysis showed that tsRNAs in CLL has been widely reported, including molecular mechanisms. ts-53, ts-42, ts-43 and ts-44 are expected to become biomarkers for the diagnosis of CLL. tRF-3019 is expected to be a potential target for the treatment of CLL.

### Prostate cancer

In 2009, Lee et al. found that the expression of ts-36 (called tRF-1001 by the author) is related to the proliferation of prostate cancer cells and promotes the transition of prostate cancer cells from the G2 phase to the M phase of the cell cycle^102^. Small RNA sequencing of prostate cancer showed that the expression levels of many tRFs in cancerous tissues and adjacent tissues were different. Most of the identified tRFs come from the 5' and 3' ends of mature cytoplasmic tRNAs. Among them, tRF-5 is the most abundant in prostate cancer cells, and most of them are upregulated. In contrast, tRF-3 dominates the downregulated tRFs. Verified by qPCR, the ratio of tRF-315 (derived from tRNALysCTT) and tRF-544 (derived from tRNAPheGAA) can effectively distinguish high- and low-grade prostate tumors and can be used as a candidate biomarker for early detection of recurrent or aggressive prostate cancer^103^. Research by Martens-Uzunova et al. showed that prostate cancer tRFs in metastatic lymph nodes are significantly increased compared with diseases that are more limited to organs, and the lengths of tRFs in nonmetastatic prostate cancer and metastatic prostate cancer samples were 18 nt and 27 nt, respectively^135^.

The above analysis shows that there are few reports on tsRNAs in prostate cancer, and the mechanism research is not in-depth. tRF-1001, tRF-315, tRF-544 and other tsRNAs are expected to be biomarkers for breast cancer diagnosis, but further studies on their molecular mechanisms are needed.

### Pancreatic cancer

Pancreatic cancer is a fatal disease with one of the highest mortality rates of all solid tumors^136^, with a 5-year survival rate of about 8%. Previously, high-throughput sequencing has identified new small noncoding RNAs. Researchers have shown that tRF may be a new biomarker for some diseases^137^. Somestudies have usedtRF and tiRNA sequences to analyze the expression levels of tRF in pancreatic cancer clinical specimens, qPCR was used to analyze the expression levels of selected tRF and tiRNA, and bioinformatics prediction was conducted to evaluate the vitalfunction of tRF and tiRNA in cancer-related pathways. Such pathways include the Ras signaling pathway, cancer pathway, and Axon guidance and PI3K/Akt signaling pathways, indicating the potential use of AS-tDR-000064 and AS-tDR-000069, whereas AS-tDR-000102 and AS-tDR-001391 could be used as diagnostic and therapeutic biomarkers for pancreatic cancer^138^. However, further studies are needed to confirm these experimental results. However, the up-regulated expression of tsRNA-ValTAC-41 in serum indicates a poor prognosis 139.

The above analysis shows that there are few reports on tsRNAs in pancreatic cancer, and the mechanism needs to be further studied. as-tdr-000064, as-tdr-000069, as-tdr-000102, as-tdr-001391 are expected to be biomarkers for the diagnosis of pancreatic cancer. tsRNA-ValTAC-41 is expected to be the targets for the prognosis and treatment of pancreatic cancer. However, further experimental studies on the molecular mechanism remain to be completed.

### Other cancers

In addition to the abovementioned cancers, recent studies have found that tRFs and tiRNAs are also related to other cancers. However, the number and depth of these studies are relatively limited, and further research is still needed.

Studies have confirmed for the first time that the expression of tiRNA-5034-GluTTC-2 is downregulated in gastric cancer tissues and plasma when compared with the normal control group and that its expression level is significantly related to tumor size. The larger the tumor diameter, the greater the expression of tiRNA-5034-GluTTC-2. The overall survival rate of patients with low expression of tiRNA-5034-GluTTC-2 is significantly lower than that of patients with high expression. These findings suggest that tRFs and tiRNAs such as tiRNA-5034-GluTTC-2 may be new potential biomarkers for the diagnosis of gastric cancer^112^.

tRF-3019a, derived from tRNA-Ala-AGC-1-1, is upregulated in gastric cancer (GC) tissues and cell lines. Phenotypic studies have shown that overexpression of tRF-3019a can enhance the proliferation, migration, and invasion of GC cells. In contrast, tRF-3019a knockdown inhibits the malignant activity of GC cells. Mechanism studies suggest that tRF-3019a directly regulates the tumor suppressor gene FBXO47. In addition, the expression level of tRF-3019a is a criterion to distinguish gastric cancer tissues from non-tumor tissues^113^. In addition, in the plasma exosomes of GC patients, the expression of tRF-25, tRF-38, and tRF-18 was higher than that of the control group, which demonstrated better accuracy in diagnosis^114^. Compared with normal liver tissues, tRF_U3_1 is most abundantly expressed in the liver cancer cell line Huh7 and cancerous liver tissues.

tRF_U3_1 is stabilized by binding La/SSB to their 3 oligo(U) orbitals and negatively regulates La/SSB-dependent viral gene expression^80^.

In the serum samples of high-grade serous ovarian cancer (HGSOC) patients and healthy controls, a total of 27 differentially expressed tRFs were identified. Differentially expressed tRFs are mainly related to protein phosphorylation, regulation of transcription and cell migration, and other functions, participating in tumor pathways, MAPK signaling pathways, FoxO signaling pathways, and Wnt signaling pathways. Among them, tRF-03357 can partially promote cell proliferation, migration, and invasion of HGSOC by downregulating HMBOX1^115^. indicating that the function of tRF-03357 in ovarian cancer may be similar to miRNA. But the specific mechanism needs further confirmation. Studies have isolated tRFs from the conditioned medium of human bladder cancer cells and confirmed that it can inhibit the growth of endothelial cells, indicating that tRF has a new role as a selective endothelial cell inhibitor in vitro^116^.

Nientiedt et al. found that the expression level of 5' tRNA4-Val-AAC is downregulated in clear cell renal cell carcinoma (ccRCC) tissues. The stage and grade of ccRCC are negatively correlated with the expression of 5' tRNA4-Val-AAC, but the exact function remains to be clarified^117^.

Martinez et al. found that compared with the control group, the serum of patients with head and neck squamous cell carcinoma (HNSCC) had significantly increased 3 5' tiRNAs(derived from tRNA-Ala, -Cys, -Tyr,) but significantly decreased 6 5' tiRNAs (derived from tRNA-Arg, -Glu, -Gly, -Lys, -Trp, and -Val)^118^.

Some researchers have screened a total of 48 tRFs from pancreatic cancer samples. Among them, the highly expressed and statistically significant tRFs and tiRNAs (AS-tDR-000064, AS-tDR-000069, AS-tDR-000102, AS-tDR-001391) were found after verification. Whether the upregulation or downregulation of these four tRFs and tiRNAs is related to the occurrence and/or progression of human pancreatic cancer still needs in-depth research in more clinical cases^138^. In addition, small RNA profiles of 80 primary uveal melanoma (UVM) samples showed high expression of tRFs in UVM. These TRFS certainly play a role in uveal cell proliferation and differentiation, but the validation of these functions requires further research^120^.

All the abovementioned differentially expressed tsRNAs play a non-negligible role in cancer progression. Only by continuously exploring their molecular mechanisms in cancer can we find more therapeutic targets.

## Conclusions and Future Perspectives

Cancer patients usually have poor prognoses, and finding ways to improve those prognoses is always a great challenge for the medical community. The exact mechanisms of progression and chemotherapeutic resistance in most cancers are unknown. tRF is a highly conserved small noncoding RNA fragment derived from tRNA. Most of TRF-5 is present in the nucleus, while most of tRF-3 and tRF-1 are present in the cytoplasm. tRFs are highly conserved, tissue-specific, time-specific, and expression stable, making themeligible as fluid-based biomarkers. This provides an easy way to study the pathogenesis and progression of cancer without the need for tissue biopsies. The study of tRFs and their target genes may help to explore new cancer diagnosis and treatment strategies, especially for some of the more aggressive subtypes. So far, the function of tRFs can be inferred from their target genes and involved pathways and analyzed with bioinformatics tools such as GO and Pathway. However, due to the limited number of studies on tRFs, not all tRFs found can be defined accurately and in detail. Perhaps with further research and expansion this deficiency will be improved. tsRNAs are functional regulatory molecules produced under specific conditions. They are abnormally expressed in lung cancer, breast cancer, colorectal cancer, liver cancer, and prostate cancer and affect tumor development to a certain extent. However, the research on tsRNAs is still in its infancy, and there are still many problems to be solved.

First of all, there is no relatively uniform system for naming tsRNAs. The human genome contains more than 500 tRNA genes. Almost all tRNAs can be cleaved by different types of ribonuclease to produce different tsRNAs. At present, conventional naming is based on the origin and type of tsRNA, but these types can only be roughly divided into two categories: tiRNA and tRF. These tsRNAs are still not classified in detail. Although some tsRNA databases simply classify and encode some tRFs according to the naming pattern of miRNAs, there is no doubt that a large number of tsRNAs have been omitted, and simple classification patterns cannot provide basic information about tsRNAs^140^.

Secondly, bioinformatics analysis shows that there are about 200 RNA nucleases that can cleave tRNAs. However, there are only a few nucleases known to cleave tRNAs, including ANG^141^, Dicer^142^, RNase P^143^,^144^, and RNase Z^102^,^145^. Therefore, a more complex network may be involved. By regulating the cleavage (and biogenesis) of tRNA, this network may require further study. Compared with other randomly degraded noncoding RNA fragments, tRFs and tiRNAs have obvious regularity. They are not randomly generated in cells but are formed through specific mechanisms. However, we know very little about the details of the generation mechanism. At present, our understanding of the mechanism of tsRNAs is mainly limited to a few specific tsRNAs. Because the human genome contains abundant tRNA genes, and the abundance of tRNA also determines the abundance of its derived fragments, there are many different types and numbers of tsRNAs. The questions of whether their expression and distribution are tissue-specific and whether their biological functions are universal or specific remain unanswered.

Thirdly, tRNAs are the most intensively modified RNA molecules with the greatest diversity of chemical modifications. These modifications determine the structure of tRNA and regulate typical tRNA functions. Recent reports indicate that these modifications are essential for regulating tsRNAs^141^. Because the current tRNA modification spectrum is limited to specific cancer cell lines, certain cell types or tissue-specific modifications must be omitted. tRNA can affect the stress-induced endonuclease activity through different modification states and then affect the cleavage process of tRNA^146^. For instance, m1A demethylated tRNA is more sensitive to the cleavage of ANG and subsequently produces tRNA-derived small RNA in the anticodon region. These tRNA-derived small RNA can enhance the assembly of ribosomes and prevent cancer cell apoptosis^147^. N6-Methyladenosine (m6A) RNA modification is a reversible epigenetic modification that has appeared in eukaryotes in recent years, not only in messenger RNA but also in noncoding RNA^148^. There are many similar tRNA modification methods; however, the potential relationship between the large number of base modifications in tRNA and the mechanism of action of tRFs and tiRNAs is currently unclear.

Finally, tsRNAs affect the tumor process to a certain extent, but whether they can be used as specific tumor markers or therapeutic targets requires in-depth study of the regulatory network between its targets, upstream molecules, and downstream molecules and technical or drug interventions on them. Furthermore, our study of tsRNAs also has some limitations. Although some tsRNAs can be used as biomarkers for clinical diagnosis and prognosis, their presence and content can only be detected by high-throughput sequencing technology and northern blot. Unfortunately, these methods cannot meet the needs of large clinical sample analysis. In addition, no matter which type of tsRNAs are derived from tRNAs, it is still difficult to specifically change the content of tsRNAs without affecting the expression level of mature tRNAs.

## Funding

This research was funded by the National Natural Science Foundation of China: No.11864002, National Natural Science Foundation of China: No.82160516.

## Figures and Tables

**Figure 1:**
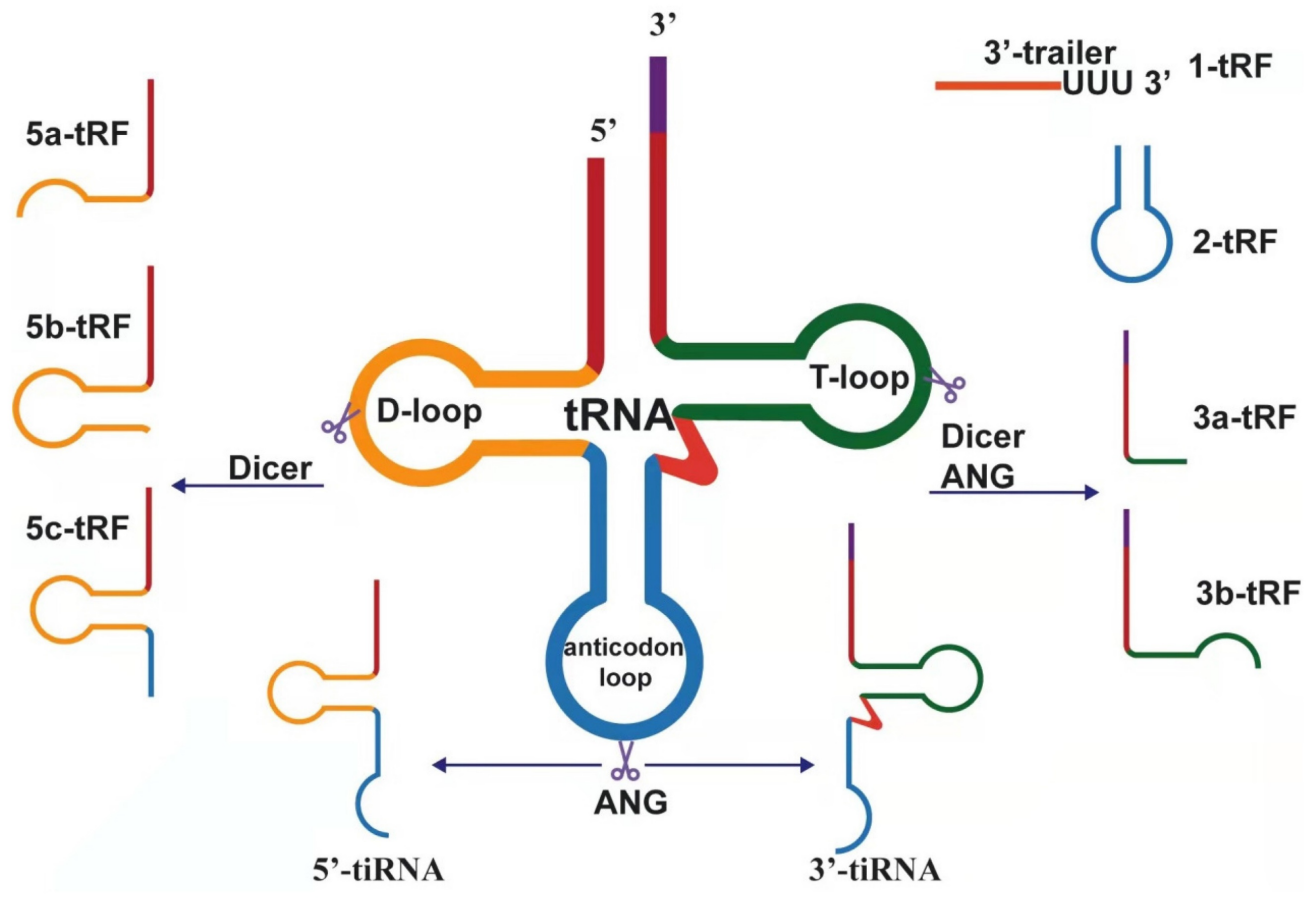
Biogenesis and classification of tsRNAs, which derived from pre-tRNA and mature tRNA.

**Figure 2:**
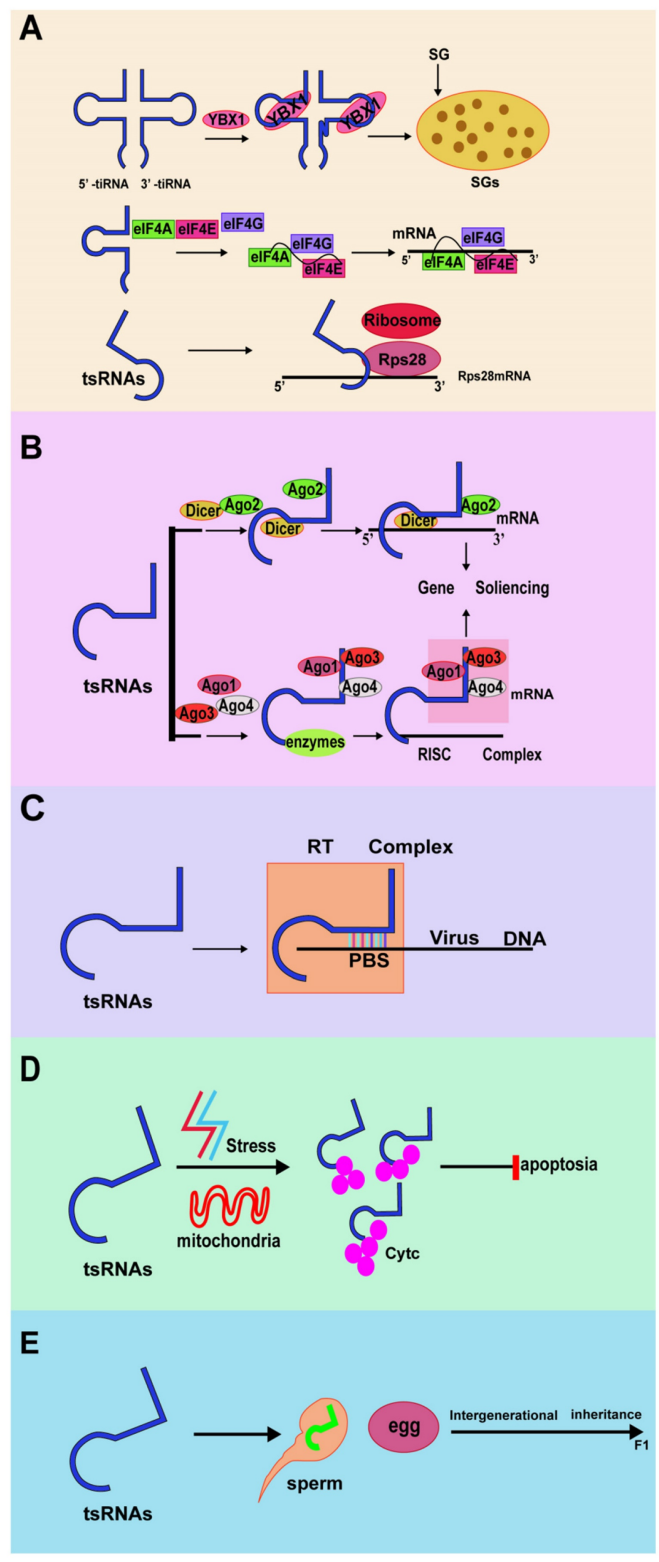
Biological function of tRNA-derived small RNAs. (A) tRFs and tiRNAs regulate translation. (B) tRFs and tiRNAs regulate gene expression. (C) tRFs and tiRNAs regulate RNA reverse transcription. (D) tRFs and tiRNAs regulate cellular activities. (E) tRFs, and tiRNAs regulate intergenerational inheritance.

**Table 1:** The role of published tsRNAs in common cancer

Cancer type	tsRNAs	Role	Reference
Breast cancer	tRFGlu, tRFAsp, tRFGly, tRFTyr	Tumor suppressor gene	37
	tRF3E	Tumor suppressor gene	95
	tDR-0009, tDR-7336	Significantly upregulated under hypoxia stimulation	96
	tsRNA-26576	Upregulated in breast cancer, performs as a potential tumor initiator and enhances tumor progression	97
	5'-tiRNAVal	Tumor suppressor gene	98
	5'-SHOT-RNA	Oncogene	99
	RUNX1-regulated ts-112	Oncogene	100
	tRF-30-JZOYJE22RR33, tRF-27-ZDXPHO53KSN	Significantly upregulated in trastuzumab-resistant breast cancer patients	101
Prostate cancer	tRF-1001/ts-36	Oncogene	102
	5'-SHOT-RNA	Oncogene	99
	tRF-315/tRFLys-CTT, tRF-544/tRFPhe-GAA	Distinguish prostate cancer grade based on their ratio	102
Colorectal cancer	tRF-1001/ts-36	Oncogene	103
	tRF/miR-1280	Tumor suppressor gene	104
	5'-tiRNA-Val	Highly expressed in CRC patients	105
Lung cancer	tRF-Leu-CAG	Oncogene	106
	ts-53/ts-3676, ts-101/ts-4521, ts-46, ts-47	Tumor suppressor gene	107,108
B-cell lymphoma	CU1276/tRF-3018	Upregulated in normal germinal center B cells	109
	AS-tDR-008946 (tRF-3) AS-tDR-013492 (i-tRF)	Upregulated and inhibit the expression of NEDD4 to accelerate lymphoma progress	110
Chronic lymphocytic leukemia	Ts-53, ts-101, ts-46, ts-47, ts43, ts-44	Downregulated in CLL patients	107,111
	tRF-3019	Found in HTLV-1 infection cells	57
Gastric cancer	TiRNA-5034-GluTTC-2	Downregulated in GC tissues and plasma	112
	tRF-3019a	Upregulated in GC tissues and cell lines	113
	tRF-18, tRF-25, tRF-38	Highly expressed in plasma exosomes of GC patients	114
Liver cancer	tRF_U3_1	Upregulated in the HCC tissues and cell lines	80
Ovarian cancer	tRF-03357	Oncogene	115
Urinary bladder carcinoma	One tRF	Isolated from the conditioned medium of human urinary bladder carcinoma cells	116
Clear cell renal cell carcinoma	5'-tiRNA	Downregulated in ccRCC tissues	117
Head and neck squamous cell carcinoma	5'-tiRNA	Differentiated expression in HNSCC samples	118
Pancreatic cancer	AS-tDR-000064, AS-tDR-000069, AS-tDR-000102, AS-tDR-001391	Highly expressed in pancreatic cancer samples	119
Uveal melanoma	tRF-5, tRF-3	Highly expressed in UVM samples	120

**Table 2:** The role of published tsRNAs in other cancers

Cancer type	tsRNAS	Role	Reference
gastric cancer	tiRNA-5034-GluTTC-2	Downregulation, promote tumor size	112
	tRF-3019a	Upregulation,directly regulates the tumor suppressor gene FBXO47,is a criterion to distinguish GC tissues from non-tumor tissues	113,114
liver cancer	tRF_U3_1	Upregulation negatively regulates La/SSB-dependent viral gene expression	80
ovarian cancer	tRF-03357	Downregulation HMBOX1, promote cell proliferation, migration, and invasion of HGSOC	115
bladder cancer	tRF	inhibit the growth of endothelial cells	116
clear cell renal cell carcinoma	5' tRNA4-Val-AAC	Downregulation, and negatively correlated with the expression of the stage and grade of ccRCC	117
head and neck squamous cell carcinoma	5' tiRNAs (derived from tRNA-Ala, -Cys, -Tyr) 5' tiRNAs (derived from tRNA-Arg, -Glu, -Gly, -Lys, -Trp, and -Val) 118	Upregulation, but it needs further research Downregulation, but it needs further research	118
pancreatic cancer	AS-tDR-000064, AS-tDR-000069, AS-tDR-000102, AS-tDR-001391	Upregulation, and are statistically significant, but it needs further research	138
Primaryuveal melanoma	tRFs	Upregulation, but it needs further research	120
